# Typical weekly physical periodization in French academy soccer teams: a survey

**DOI:** 10.5114/biolsport.2023.119988

**Published:** 2022-10-14

**Authors:** Tom Douchet, Christos Paizis, Christopher Carling, Carole Cometti, Nicolas Babault

**Affiliations:** 1 INSERM UMR1093-CAPS, Université Bourgogne Franche-Comté, UFR des Sciences du Sport, F-21000, Dijon; 2 Centre d’Expertise de la Performance, Université Bourgogne Franche-Comté, UFR des Sciences du Sport, F-21000, Dijon; 3 Dijon Football Côte d’Or (DFCO), 17 rue du Stade, 21000 Dijon, France; 4 Fédération Française de Football (FFF), Paris, France

**Keywords:** Physical development, Microcycle, Youth, Strength, Aerobic, Speed

## Abstract

In elite-level youth soccer players, weekly training periodization is of paramount importance to plan for short- and long-term physical development. The present study investigated current practices for physical periodization strategies in elite male French academies. An online survey was completed by elite French academies strength and conditioning coaches to determine the typical weekly periodization with particular reference to daily training in relation to match day (MD) in youth soccer players. The survey attempted to characterize the importance of physical development compared to match result, and practices used (expected difficulty and content) for each training session according to duration, exercises, and objective. The frequency rates of the responses were compared using two-tailed Chi-square tests with the significance level set at p < 0.05. Fortyfive questionnaires were analyzed. Respondents indicated that their training sessions focused mainly on physical development (95.6%) rather than match result. Active recovery (34.2%) and aerobic conditioning exercises (40.8%) were primarily conducted on MD+1 and MD+2 using passing circuits and aerobic technical drills. Physical development was mostly pursued during sessions on MD-4 (38.8%) and MD-3 (37.3%). The number of large-sided games was highest on MD-3 (58.1%). On MD-2 and MD-1, a decrease in the training load was highlighted, with speed (40.4%) and tapering sessions (52.4%) mostly implemented. Intensive use of small-sided games (92.3%) and reactivity exercises was observed at MD-1 (100.0%). Our results revealed discrepancies between the physical objectives set for each day and the content implemented, which could potentially be more physically demanding than expected.

## INTRODUCTION

Effective training periodization within the academy systems is key for developing elite soccer players and ensuring that they are prepared for future demands at professional standards. In elite academy settings, young players usually train three to seven times a week, with the greatest workload being the competitive match at the end of the week [1, 2]. Weekly periodization, here referred to as a ‘microcycle’, is of paramount importance for short and long-term player development and performance [3]. Unfortunately, the multifactorial approach to training in soccer academies can render difficult the implementation of any periodization strategy. Indeed, academy staff must ensure players’ educational, physical, technical, and tactical development while ensuring they perform maximally during the weekly competitive games. Furthermore, balancing these double aims must be considered alongside management of other associated factors such as fatigue, recovery [4, 5], and injury prevention [6].

Currently, research describing the in-season weekly training periodization of young soccer teams is fairly limited [2, 5, 7, 8]. Studies have shown that external loads were progressively increased up to three days before match day (MD-3), and decreased the day preceding the match (MD-1) [5, 7]. In U17 players belonging to a Portuguese academy, the lowest external load was observed at MD-1 [5]. Sprinting frequency was notably 7.4% and 19.0% less than post-match or mid-week, respectively. In the same study, in U19 players, the difference was even greater with the number of sprints almost 90% lower than in post-match or mid-week sessions. In contrast, unclear differences have generally been observed in measures of training loads between post-match and mid-week sessions [5]. For example, in an English Premier League academy [2], the greatest training load was obtained at MD+2. A reasonable explanation for this result is that the players participated in double training sessions inevitably increasing their daily training load, while it is almost never the case for professional teams [9].

While the above studies in academy settings highlight day-to-day differences in training load over a typical weekly periodization period, to the best of our knowledge, only one study has described the typical training content on a day-to-day basis [8]. Briefly, an academy team was firstly shown to rest on MD+1. It then performed development/maintenance training of aerobic capacity on MD+2, development/maintenance of aerobic power and/or maximal power on MD-4, position-specific training on MD-3, team tactical organization on MD-2, and development/maintenance of reactivity and acceleration on MD-1. Such a periodization strategy would suggest that practitioners emphasize players’ development over match result.

More generally, research is needed to confirm this in all elite French academies rather than in a single team and identify the workload strategies. This different approach to data collection could allow the whole practice to be compared with the practices of academies in other countries.

Similarly, although previous studies have arguably provided useful insights into weekly periodization amongst academy teams, studies have mostly been conducted in single club settings. Furthermore, key information on physical training content (speed, strength or aerobic) is lacking during the microcycle. Similarly, information specifically relating to the exercises used to enhance these physical qualities is unavailable. Accordingly, the present study aimed, through an online survey, to determine the typical microcycle periodization (expected difficulty and content) prior to a competitive weekend game in multiple French elite male soccer training academies. Particular focus was given to determining the content of each daily training session. We also hypothesized that the academies would be primarily concerned with the technical and physical development of their players rather than match result.

## MATERIALS AND METHODS

### Subjects

Strength and conditioning (S&C) coaches from 36 elite French academies were invited via email to complete the online survey concerning their U17 and U19 teams. Unanswered questions were excluded from the analyses. Data were collected from the beginning of October to the end of November 2020. All responses were confidential and anonymized. All respondents were notified of the research protocol, benefits and risks before providing consent in accordance with the declaration of Helsinki. Approval of the study was obtained from the local ethics committee. Respondents were clearly informed that their consent was obtained by responding to the first question of the survey.

### Procedures

The survey was developed in French and administered via an online survey software (Google Form). The number of questions depended on the number of sessions performed by the team. As such, practitioners had to respond to a number of questions ranging from 17 (only mandatory questions and no training session at all), to 179 (mandatory questions and 3 sessions per day for 6 days). The survey included multiple-choice, close-ended and open-ended questions (depending on the answers submitted, respondents had to respond to additional questions to provide further information when necessary) (Table S1). The survey first described key information about the study, its purpose as well as information related to the research team associated with an e-mail contact. Respondents were then asked to describe the competitive week. For inclusion, the selected week should have been preceded and followed by a competitive match (7-day interval between each competitive match).

Questions covered four main themes: 1) participant information; 2) their subjective rating on the overall importance of physical development compared to the results of competitive matches (1 to 10 scale: 1 = maximum importance of the match result); 3) description of each training session from MD+1 (first day after match day) to the following MD (match day) with particular reference to the physical conditioning work performed (duration, physical quality focus, integrated or dissociated exercises, exercises, expected difficulty), and 4) periodization improvements that could be operated to increase players’ physical development. The design of the survey was based on extensive discussions, suggestions and feedback between the research team and practitioners. The content of the final version of the questionnaire was validated by calculating the content validity index (CVI). Eight experts (including coaches and scientists) were requested to rate the relevance of the different items questioned. The content of the present survey was validated with an average scale-CVI greater than 0.89 [10]. The survey was subsequently pilot-tested in five French soccer academy teams to review content clarity prior to the sending of official invitations. This process removed two questions, merged four questions into two, and several rewordings were made for clarity [11].

### Statistical analyses

Data were first reviewed and any incomplete responses subsequently excluded from the analyses. The distribution of the response frequencies was analyzed. Descriptive statistics are reported in the form of counts out of the total number of answers for the question. The frequency distribution between days was compared using two-tailed Chi-square tests with the significance level set at p < 0.05. In the event of a significant Chi-square, pairwise comparisons were achieved using a Z-test. Repeated measures ANOVA, with Bonferroni’s post hoc test were performed to determine differences between days for physical importance and expected difficulty of the sessions. Statistics were performed using JASP (Ver 0.13, JASP Team (2020), University of Amsterdam) and SPSS (Ver 27, IBM-SPSS Inc., Armonk, NY, USA). Open-ended responses were arranged and read several times to ensure an understanding of their meaning. The list of themes of the open responses was discussed by all researchers until consensus was reached regarding data interpretation and theme credibility [12].

## RESULTS

### Response rate

A total of 50 responses were obtained out of a possible 72 which represent a 69.4% survey return rate. Of these, 45 were included in the analyses (Table S2) while 5 surveys were excluded owing to incomplete responses. These numbers correspond to a 90.0% completion rate and 10.0% omission. These numbers are in line with survey papers in the soccer literature [13, 14].

### Training session information

**FIG. 1:**
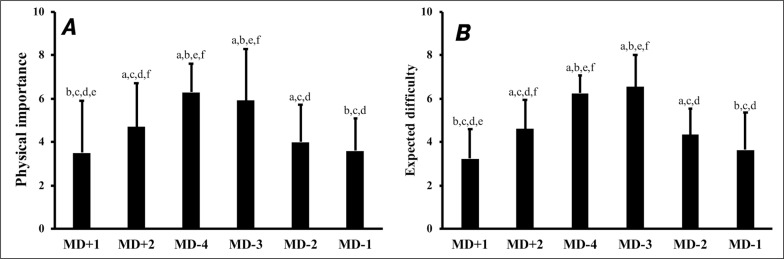
(A) Physical importance, and (B) Expected difficulty, for each day (/10). Significant differences between days according to repeated measures ANOVA are shown for p < 0.05 from a: MD+1; b: MD+2; c: MD-4; d: MD-3; e: MD-2; f: MD-1. MD: match day.

Respondents rated the overall perceived importance of physical development relative to the match result as 7.3 ± 1.6 out of 10 (1 = maximum importance of the match result). The large majority of respondents (95.6%) prioritized players’ physical development over competitive match results. The physical importance (p < 0.001) and training expected difficulty (p < 0.001) varied between-days. Both variables increased progressively until MD-3 before a reduction was observed on MD-2 and MD-1. No difference occurred between MD-4 and MD-3 (Figure 1).

The number of training sessions was different between days (p < 0.001) (Table 1). Most teams did not train at MD+1. A single training session was conducted daily at MD+1, MD+2, MD-2, MD-1. Two training sessions per day were generally conducted on MD-4 and MD-3. Training duration also differed between days (p = 0.041) (Table 1). Training lasted predominantly between 30 and 60 min on MD+1 and MD-1. On the remaining days, most training sessions lasted between 60 and 90 min.

**TABLE 1:** Information on training sessions.

	MD+1	MD+2	MD-4	MD-3	MD-2	MD-1
**Number of training sessions a day (number of responses / total number of responses)**						
0 training session***	27/45^b,c,d,e,f^	0/45^a,e,f^	0/45^a,e,f^	0/45^a,e,f^	5/45^a,b,c,d^	4/45^a,b,c,d^
1 training session***	18/45^b,c,d,e,f^	32/45^a,f^	23/45^a,e,f^	31/45^a,f^	37/45^a,c^	41/45^a,b,c,d,e^
2 training sessions***	0/45^b,c,d,e^	13/45^a,e,f^	22/45^a,e,f^	14/45^a,e,f^	3/45^a,b,c,d,f^	0/45^b,c,d,e^

**Duration of the session (number of responses / total number of session)**						
Up to 30 min*	0/18	0/58	1/67	3/59	0/43	0/41
More than 30 min and up to 60 min***	17/18^b,c,d,e^	23/58^a,c,d,e,f^	14/67^a,b,e,f^	13/59^a,b,e,f^	18/43^a,b,c,d,f^	31/41^b,c,d,e^
More than 60 min and up to 90 min***	1/18^b,c,d,e^	29/58^a,f^	33/67^a,f^	33/59^a,f^	24/43^a,f^	10/41^b,c,d,e^
More than 90 min***	0/18^c^	6/58^c,f^	19/67^a,b,e,f^	10/59^e,f^	1/43^c,d^	0/41^b,c,d^

Values are presented as the number of responses and percentage of the total number of answers (n) for number of training sessions and duration. Significant frequency distribution between days with respect to Chi-square for

* = p < 0.05;

** = p < 0.01;

*** = p < 0.001. Significant differences between days according to Z-score for p < 0.05 are shown from

a : MD+1;

b : MD+2;

c : MD-4;

d : MD-3;

e : MD-2;

f : MD-1. MD: Match day.

**FIG. 2:**
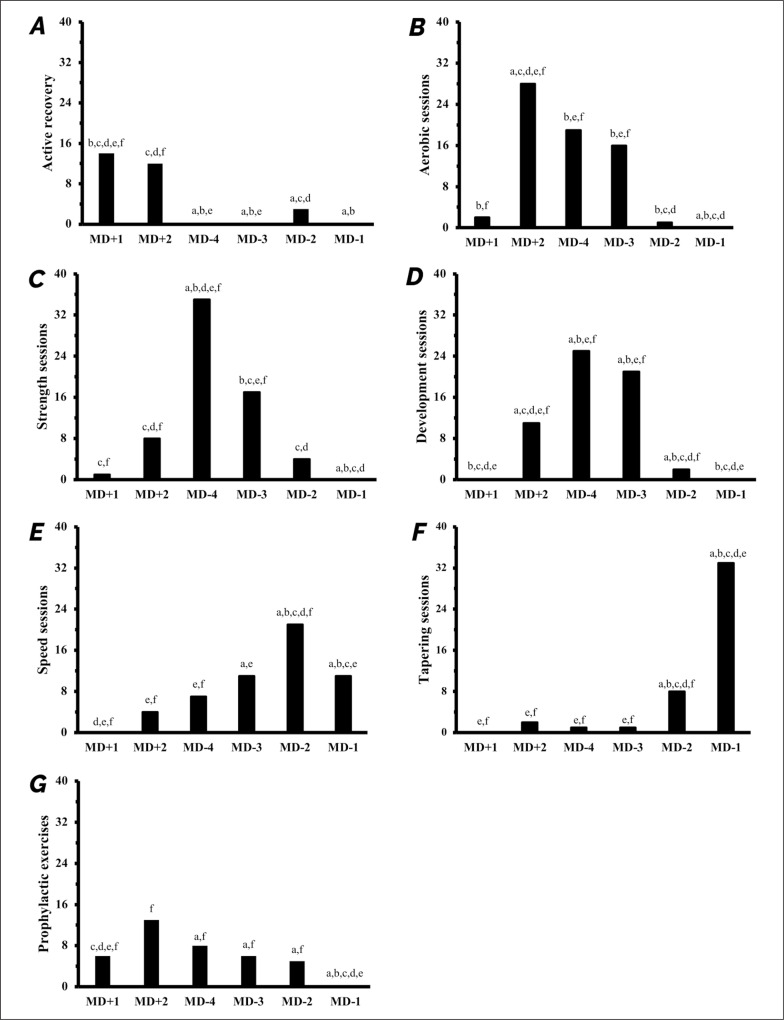
Number of training sessions through the week for (A) active recovery sessions, (B) aerobic training sessions, (C) strength training sessions, (D) development training sessions, (E) speed training sessions, (F) tapering sessions, (G) prophylactic exercises. Results represent number of responses for each day. Significant differences between days according to Z-scores are shown for p < 0.05 from a: MD+1; b: MD+2; c: MD-4; d: MD-3; e: MD-2; f: MD-1. MD: match day.

Differences were observed between days for physical quality focus (p = 0.002) (Figure 2). MD+1 and MD+2 were mainly characterized by active recovery (Figure 2A). Aerobic sessions were usually performed from MD+2 to MD-3 (Figure 2B) while strength sessions were predominantly conducted during MD-4 and MD-3 (Figure 2C). The frequency of speed training sessions gradually increased until MD-2 and was prominent on MD-2 and MD-1 (Figure 2E). Tapering sessions were implemented on MD-2 and MD-1 (Figure 2F). While prophylactic exercises were mostly performed on MD+2, these were performed evenly throughout the week except for MD-1 (Figure 2G).

Both integrated and dissociated training types were used for physical conditioning training. Integrated physical content included different exercises with varying frequencies during the week (p = 0.028) (Table 2). Passing circuits, alongside aerobic technical drills, were mostly practiced during MD+2, while large-sided games were mainly used on MD-3. However, no differences were observed during the week for small-sided games (p = 0.180), medium-sided games (p = 0.085), rondo (p = 0.508), technical drills (p = 0.735), or football tennis (p = 0.059). Similarly, the frequency of dissociated physical contents varied during the week (p = 0.020) (Table 2). Continuous aerobic exercises were exclusively performed on MD+1 and MD+2. Upper-body strength sessions were mainly used from MD+1 to MD-3. On MD-4 and MD-3, practitioners primarily implemented lower-body strength sessions, intermittent aerobic, repeated-sprint aerobic, and plyometric exercises. Speed and reactivity exercises were mostly used on MD-2 and MD-1. The use of coordination exercises did not show any significant differences between days (p = 0.149).

**TABLE 2:** Physical quality focus of the training sessions.

	MD+1	MD+2	MD-4	MD-3	MD-2	MD-1
**Integrated exercise(s) $ (number of responses / total number of integrated session)**						
Small-sided games	2/4	14/34	20/29	12/31	4/23	12/13
Medium-sided games	2/4	16/34	9/29	13/31	9/23	2/13
Large-sided games***	2/4^f^	4/34^d^	5/29^d^	18/31^b,c,e,f^	7/23^d,f^	0/13^a,d,e^
Rondo	0/4	6/34	4/29	8/31	5/23	4/13
Technical drills	2/4	11/34	8/29	9/31	6/23	5/13
Passing circuit*	2/4	18/34^c,d^	6/29^b,f^	6/31^b,f^	8/23	7/13^c,d^
Football tennis	2/4	4/34	0/29	1/31	2/23	0/13
Aerobic technical drills*	0/4	5/34^c^	0/2^b^	1/31	1/23	0/13

**Dissociated exercise(s) $ (number of responses / total number of dissociated session)**						
Upper-body strength session**	5/12^e,f^	5/2^f^	11/32^f^	6/25^e,f^	1/14^a,d^	0/27^a,b,c,d^
Lower-body strength session***	0/12^b,c,d^	6/21^a,f^	15/32^a,e,f^	10/25^a,e,f^	1/14^c,d^	0/27^b,c,d^
Speed*	0/12^d,e,f^	3/21^e^	4/32^e^	7/25^a,e^	10/14^a,b,c,d,f^	5/27^a,e^
Reactivity***	0/12^e,f^	0/21^e,f^	0/32^e,f^	0/25^e,f^	4/14^a,b,c,d,f^	27/27^a,b,c,d,e^
Continuous aerobic exercises***	8/12^b,c,d,e,f^	6/21^a,c,d,e,f^	0/32^a,b^	0/25^a,b^	0/14^a,b^	0/27^a,b^
Intermittent aerobic exercises*	3/12^e,f^	6/21^e,f^	10/32^e,f^	10/25^e,f^	0/14^a,b,c,d^	0/27^a,b,c,d^
Repeated-sprint aerobic exercises***	1/12^d^	2/21^d^	8/32^e,f^	12/25^a,b,e,f^	0/14^c,d^	0/27^c,d^
Plyometric*	0/12^c,e^	3/21^c^	14/32^a,b,f^	5/25	4/14^a^	2/27^c^
Coordination	5/12	7/21	7/32	3/25	5/14	7/27

**Duration of the dissociated contents (number of responses / total number of dissociated session)**						
Up to 20 min***	4/12^e,f^	8/21^e,f^	17/32^e,f^	12/25^e,f^	12/14^a,b,c,d,f^	27/27^a,b,c,d,e^
More than 20 and up to 40 min***	7/12^c,e,f^	7/21^e,f^	8/32^a,e,f^	10/25^e,f^	0/14^a,b,c,d^	0/27^a,b,c,d^
More than 40 and up to 60 min	1/12	5/21	6/32	3/25	2/14	0/27
More than 60 min	0/12	1/21	1/32	0/25	0/14	0/27

**Dissociated exercises (number of responses / total number of dissociated session)**						
Field-based session***	9/12^f^	12/21^f^	21/32^f^	17/25^f^	12/14^f^	27/27^a,b,c,d,e^
Gym-based session***	3/12^f^	9/21^f^	11/32^f^	8/25^f^	2/14^f^	0/27^a,b,c,d,e^

Values are presented as the number of responses and as the percentage of the total number of answers (n) for the question for each day as reported below the question. $ Questions with the possibility to give multiple responses. Significant frequency distribution between days with respect to Chi-square for

* = p < 0.05;

** = p < 0.01;

*** = p < 0.001. Significant differences between days according to Z-score for p < 0.05 are shown from

a : MD+1;

b : MD+2;

c : MD-4;

d : MD-3;

e : MD-2;

f : MD-1. MD: Match day.

The duration of the dissociated contents differed through the week (p < 0.001) (Table 2). Sessions lasting more than 20 min and up to 40 min were used more frequently from MD+1 to MD-3. Sessions lasting up to 20 min were mostly used from MD-4 to MD-1. However, no differences were obtained for the few sessions lasting longer than 40 min.

## DISCUSSION

In this survey, the results partly confirmed our hypothesis that in theory academies prioritized physical development over match results. However, in reality training sessions specifically dedicated to physical development were only observed on two days (MD-4 and MD-3). Results demonstrated that teams mainly performed recovery-oriented sessions on MD+1 and MD+2 and tapering sessions on MD-2 and MD-1. The novelty of this study is that it is the first study to describe the periodization of weekly training for all elite academies in a country. It therefore appears that training periodization was only partly congruent with the expected training objective.

On MD+1, responses from the present survey showed that recovery was the primary physical aim, with the implementation of different recovery strategies (active or passive). Research examining physical, physiological and perceptual markers has shown that recovery following a soccer match is incomplete on MD+1 [15]. As such, active recovery should be [16] and was implemented by academy practitioners [17, 18] during MD+1, no doubt in an attempt to aid the recovery process. Indeed, the intensity of the MD+1 session was the lowest of the week. Results showed that training duration was increased by the coaching staff on MD+2. Additionally, a large number of sessions lasting at least 60 min, and double sessions were reported on this day. In line with our results, a previous study also reported greater training loads on MD+2 [19]. In contrast, findings are not consistent as other studies reported lower training volumes in both professional [17] and youth teams [18], while some reported the greatest weekly training load on MD+2 [2]. These discrepancies across results could be related to contextual variables such as the number of training sessions on MD+2 (e.g., 2 training sessions [2] vs. 1 training session [17]) or training content (implementation of strength training [2]).

The training content on MD+2 demonstrated a preference for small- and medium-sided games rather than large-sided games. This choice could be explained by practitioners accounting for the highest values of creatine kinase that typically occur on MD+2 resulting from the previous match, which are associated with muscle damage [20] and delayed onset muscle soreness [21]. Indeed, small- and medium-sided games have been shown to be a more suitable option to ensure lower overall physical (external load) demands in comparison to large-sided games [22]. However, depending on the format and particularly the number of players involved, in relation to the area per player available [23], external loading in small- and medium-sided games could potentially be more intensive than peak periods observed in official match-play format particularly in relation to the frequencies of accelerations and decelerations and player load [24]. Rules can also impact the physiological demands of small-sided games [25]. Accordingly, the number of players (i.e., area per player) and rules should be adjusted to ensure that the physiological impact of these training sessions is not too intensive. Future research should examine whether this is the case or not across academies.

Here, the greatest training load was reported on MD-4 and MD-3. The external loads experienced by players on these days (farthest away from match-day) usually aim to replicate competitive demands [17, 26]. As such, practitioners reported the greatest expected difficulty, and number of physical development sessions during these two days. Increases in dissociated training contents were observed during these two days with the greatest number of lower-body strength sessions, intermittent aerobic, repeated sprint, and plyometric exercises. Hence, it would seem that the dissociated training contents were perceived as more efficient than integrated contents to develop players’ physical qualities.

Practitioners often reported using small-sided games on MD-4. Small-sided games have been shown to induce greater neuromuscular constraints (i.e., accelerations and decelerations) than other forms of integrated training [27, 28]. During this day, the amount of strength training reported by practitioners was greater compared to the other days, but was still very low. Indeed, our results highlighted that very few lower-body strength and plyometric sessions (33.3% and 31.1% of the practitioners, respectively) were programmed. Dissociated strength and plyometrics training contents should be emphasized further to favor neuromuscular development. It is well known that these dissociated exercises impose high neuromuscular stress that obviously favor strength development [29, 30].

During MD-4 and MD-3, coaches reported using aerobic development sessions. For this, both dissociated (intermittent and repeated-sprint aerobic exercises) and integrated training contents were used to develop aerobic quality. Such exercises appear in accordance with findings in the literature. A previous study for example, demonstrated that integrated contents can result in comparable aerobic improvements to dissociated contents [31].

Speed development training was only occasionally reported. To train this component, practitioners prioritized integrated contents with large-sided games. This choice can be legitimized by the greater high intensity demands imposed by large-sided games as compared to other integrated training forms [28]. However, as for strength development, implementing dissociated speed situations could optimize training efficiency through better control of sprinting exercises (e.g., a set number of sprints) [32].

A decrease in the training load (expected difficulty and physical importance) was observed two days prior to the match (MD-2 and MD-1). This is highlighted by the almost complete absence of voluminous and double sessions per day. On MD-2 and MD-1, speed and tapering were the primary focuses. This tapering method is commonly observed in soccer during the final two days leading up to competition [2, 5, 33, 34]. On MD-2, practitioners reported an increased use of medium- and large-sided games and dissociated speed sessions. The implementation of large-sided games and dissociated speed training are a mismatch with the expected training objectives. Indeed, these sessions might favor physical development over tapering as a large amount of high speed running can lead to neuromuscular impairments still observable 48 h post training intervention [35]. Reduced distances [33] should be favored to limit unexpected impairments potentially detrimental for the subsequent match.

On MD-1, most coaches used similar content. Small-sided games and reactivity drills were extensively performed. Previous studies also report the use of reactivity drills close to the competition [8, 19]. Small-sided games do not involve significant demands for high-speed running [28]. We could hypothesize that high-speed running is reduced to limit excessive demands and reduce delayed onset of fatigue. However, small-sided games do result in a large number of accelerations and decelerations [28, 36] potentially resulting in neuromuscular impairments [22]. The present results are not consistent with previous findings for MD-1 since most studies reported light training loads [4, 17, 37], and low-load exercises [17]. The contents implemented during MD-2 and MD-1 do not appear to support the tapering strategy aimed for by practitioners.

### Limitations

Limitations in the present study include potential bias related to the coaches’ willingness to share information. Furthermore, this study only solely reflects the convenience of the teams’ consideration and therefore their strength and conditioning coaches, which is a inherent bias of research of this nature. This survey aimed to describe the weekly collective periodization strategy and as a result, individualized sessions were not considered. It would have been of interest to precisely determine training workload through the week using external and internal measures such as GPS and HR. Moreover, although a substantial number of teams (n = 45) completed the present survey, additional responses would potentially have increased knowledge of academies’ weekly periodization strategies. Additional respondents, and from different countries, would have increased the opportunities to identify different strategies for optimal conditioning for match-play.

### Practical applications

The present survey revealed that coaches placed more emphasis on physical development than match result. Yet, this result is contrain-dicatory since only two training sessions per week were dedicated to physical development. MD+1 and MD+2 were mainly dedicated to recovery, and MD-2 and MD-1 to tapering. This type of periodization is comparable to professional teams that mainly focus on competition rather than development. To favor player development, we propose to implement more dissociated contents. Dissociated strength sessions should be performed more than once a week to generate larger improvements [38, 39]. We also propose to change MD-1 training contents with more tactical contents to support the tapering strategy and potentially ensure a better level of readiness on MD.

## CONCLUSIONS

This study showed that although emphasis was placed upon physical development by practitioners, in reality this was only really performed over two days (MD-4 and MD-3). Our results revealed discrepancies between the physical objectives set for each day and the content actually implemented, which potentially could be more physically demanding than expected. Further studies should clarify the fatigue caused by each training session to ensure that training periodization matches practitioners’ expectations.

## Supplementary Material

Typical weekly physical periodization in French academy soccer teams: a survey
