# Supplementary material for: Typical weekly physical periodization in French academy soccer teams: a survey
Source: Biol Sport. 2022 Oct 14;40(3):731–40. doi: 10.5114/biolsport.2023.119988 (PMC10286623; doi:10.5114/biolsport.2023.119988)
Supplement: Typical weekly physical periodization in French academy soccer teams: a survey [file JBS-40-119988-s1.pdf]

## SUPPLEMENTARY TABLES

**TABLE S1.** Questionnaire used during the present study.

| Questions | Responses | Other information |
|---|---|---|
| Q1 – By clicking 'Yes', you give your informed consent to your voluntary participation. | Yes, No | |
| Q2 – How important is the players' physical development in comparison to matches' results? | Linear scale from 1 (not important at all) to 10 (very important) | |
| Q3 – Do you realise a training session on this day? | Yes, No | Duplicate question if additional training/ day – Close-ended question |
| Q4 – What is the duration of the session? | Up to 30 min, More than 30 min and up to 60 min, More than 60 min and up to 90 min, More than 90 min | Duplicate question if additional training/ day – Close-ended question |
| Q5 – How much physical work is done during the training session? | Linear scale from 1 (no physical work at all) to 10 (only physical work) | Duplicate question if additional training/ day – Close-ended question |
| Q6 – What is(are) the physical aim(s) of the training session? | No physical aim, Active recovery, Tapering, Maintenance, Development, Speed, Aerobic, Strength, Prophylactic work | Duplicate question if additional training/ day – Close-ended question<br>* Multiple responses |
| Q7 – How is practiced the main physical work? | Integrated, Dissociated on the field, Dissociated outside of the field, No physical work | Duplicate question if additional training/ day – Close-ended question |
| Q8 – Which exercise(s) is(are) used during this session? | Small sided games, Medium sided games, Large sided games, Rondo, Technical drills, Passing circuit, Football tennis, Aerobic technical drills, Others | If Q7 is 'Integrated'<br>Duplicate question if additional training/ day – Close-ended question<br>* Multiple responses |
| Q9 – Which exercise(s) is(are) used during this session? | Upper body strength session, Lower body strength session, Speed, Reactivity, Continuous aerobic work, Intermittent aerobic work, Repeated sprint aerobic work, Plyometric work, Coordination, Others | If Q7 is 'Dissociated on the field' or 'Dissociated outside of the field'<br>Duplicate question if additional training/ day – Close-ended question<br>* Multiple responses |
| Q10 – What is the duration of this work? | Up to 20 min, More than 20 min and up to 40 min, More than 40 min and up to 60 min, More than 60 min | If Q7 is 'Integrated'<br>Duplicate question if additional training/ day – Close-ended question |
| Q11 – What is the expected difficulty of this session? | Linear scale from 1 (very very easy) to 10 (maximal) | Duplicate question if additional training/ day – Close-ended question |
| Q12 – Do you practice another training session on this day? | Yes, No | Duplicate question if additional training/ day – Close-ended question |
| Q13 – In your opinion, what changes could you operate in your weekly periodization, in order to optimize the players' physical development, and without negatively impacting match performance? | | Open-ended question |
| Q14 – What is the level of your team? | Academy, Professional, Amateur | Close-ended question |
| Q15 – What is the gender of the players? | Female, Male | Close-ended question |
| Q16 – Which category do you coach? | Adults, U18/U19, U16/U17, Others | Close-ended question |
| Q17 – What is your role inside the team? | Coach, Strength and conditioning coach, Others | Close-ended question |
| Q18 – What is your education? | Bachelor, Master's degree, Federal education, University diploma, Others | Close-ended question |
| Q17 – How long have you been coaching? | Up to 1 year, More than 1 year and up to 3 years, More than 3 years and up to 5 years, More than 5 years and up to 10 years, More than 10 years | Close-ended question |

**TABLE S2.** Respondents' information.

| | |
|---|---|
| **Gender** | |
| Male | 45 (100%) |
| Female | 0 (0%) |
| **Role** | |
| Coach | 1 (2.2%) |
| Strength and conditioning coach | 44 (97.8%) |
| Other | 0 (0%) |
| **Education $** | |
| Bachelor | 8 (10.1%) |
| Master degree | 31 (39.2%) |
| Federal education | 18 (22.8%) |
| Other diploma from University | 20 (25.3%) |
| Other | 2 (2.5%) |
| **Experience** | |
| Up to 1 year | 0 (0%) |
| More than 1 year and up to 3 years | 8 (17.8%) |
| More than 3 years and up to 5 years | 12 (26.7%) |
| More than 5 years and up to 10 years | 17 (37.7%) |
| More than 10 years | 8 (17.8%) |

Values are presented as the number of responses and as the percentage of the total number of answers for the question. Questions with the possibility to give multiple responses ($).

**TABLE S3.** Periodization improvements.

| **In your opinion, what changes could you operate in your weekly periodization, in order to optimize the players' physical development, and without negatively impacting match performance? $** | |
|---|---|
| **Number of answers for the question** | **45** |
| Change the session schedule | 3 (6.7%) |
| Add lower body strength sessions | 4 (8.9%) |
| Improve training load monitoring | 2 (4.4%) |
| Add more sessions / increase training volume | 11 (24.4%) |
| Decrease the number of sessions | 1 (2.2%) |
| Change the physical aim of the days | 4 (8.9%) |
| Add dissociated work | 1 (2.2%) |
| Improve individualisation | 3 (6.7%) |
| Add a training the morning preceding the match | 2 (4.4%) |
| Improve tapering close to the match | 2 (4.4%) |
| No possibility | 1 (2.2%) |
| No response | 11 (24.4%) |

Values are presented as the number of responses and as the percentage of the total number of answers (n) for the question as reported below the question. Answers were grouped according to their similar nature.

**TABLE S4.** Typical microcycle.

| Day | Description |
|---|---|
| MD+1 | Recovery or One 30–60 min training session – Active recovery – Continuous aerobic work – Upper body strength session |
| MD+2 | One 60–90 min aerobic development session – Passing circuit – Small- medium-sided games |
| MD-4 | One/two 60–90 min strength development sessions – Small- sided games – Upper/lower body strength sessions – Plyometric exercises |
| MD-3 | One 60–90 min aerobic/strength development session – Large-sided games – Repeated sprint aerobic exercises |
| MD-2 | One 60–90 min speed session – Small- and medium-sided games – dissociated speed – reactivity |
| MD-1 | One 30–60 min tapering session – small-sided games – reactivity |
